# Bio-objects and generative relations

**DOI:** 10.3325/cmj.2012.53.198

**Published:** 2012-04

**Authors:** Sakari Tamminen, Niki Vermeulen

**Affiliations:** 1 Department of Social Research, University of Helsinki, Helsinki, Finland sakari.tamminen@helsinki.fi; 2 Centre for the History of Science, Technology and Medicine, University of Manchester, Manchester, UK niki.vermeulen@manchester.ac.uk; On behalf of the members of Working Group 3 “Bio-objects and Generative Relations” of the COST Action “Bio-objects and their Boundaries: Governing Matters at the Intersection of Society, Politics and Science”, see http://www.univie.ac.at/bio-objects

Working Group 3 of the COST Action IS1001 examines bio-objects and their generative relations. This group and its work seek to transcend disciplinary boundaries in humanities, social sciences, and life science and mingle with policy research as well. It also aims to envelop and synthesize various strands of research by taking life science and its relations as subject matter while crossing borders between research settings.

One of the key reasons for this networked collaborative approach of Working Group 3 is the scale and nature of previous efforts investigating the development of the life sciences. Researchers in humanities and social sciences, regardless of their collaboration networks, are still far from large scale collaboration when imagining the future of our collective life alongside living *objects* produced in the technoscientific processes of today. And while there are highly interesting current approaches to studying transformations in understandings of life, they are found among disciplines within human and social sciences that do not interact sufficiently with each other. Philosophy (of biology and bio-ethics), anthropology (of science and medical communities), sociology (of science), political science (of institutional sense-making and deliberation), and legal studies (of jurisdictions), to name but a few, have their own lives in specific epistemic communities of practice. What we do know already is that when we want to think about life – as a vitalist notion, as a biochemical process, as a mechanistic system, or as the force underlying a social system and its politics – we immediately step into a field of competing discourses, framing “life” as an object of representation, intervention, and manipulation in a myriad of ways.

In parallel with these academic debates, recent advances in the biological sciences, including new medical technologies, have, however, also led to the analysis of various transformations in the process and understanding of life. These are already articulated with/in diverse bodies – corpora from the literature; organic matter and its networks of circulation; and more “stable” institutions of economic, legal, or political import – with different effects. It appears that bio-objects (1) are rich in potential to destabilize old relations and fertile enough to create new connections that cross the boundaries of academic disciplines and between social institutions.

The Working Group 3 has a unique opportunity to address the challenges outlined above and has a two-part main objective: 1) tracking new experimental relations that bio-objects bring about by 2) weaving relations among scholars otherwise unconnected to each other. Accordingly, the group not only investigates the relations that new objects of living and life are capable of generating but also attempts, with a more reflexive attitude, to become more experimental in its ways of working to address the challenges posed by bio-objects (Box 1).

Box 1 **Our work is characterized by three dimensions in the forming of new relations in the study of bio-objects**

1. Global networks: The group attempts to go beyond single case studies in specific national contexts, by developing coherent international comparative frameworks built around the concept of the bio-object. We specifically aim to ground our international comparative framework in detailed local empirical work in which matters of life and living together play an important role. Consider this a call for collaborative research.

2. Common ground: By using the concept of bio-objects as a call for collaboration and, thereby, as a network-generating device, in our studies we explicitly focus on a wide range of experimental relations that are empirically traceable in different contexts. These include material, scientific, social, cultural, economic, and political relations embedded in processes by which bio-objects are becoming a central part of the relations that go into the everyday politics of living together in the 21st century.

3. Informative function: In line with the two dimensions outlined above, we also deliberately aim to cross the borders of the academic community, making our bio-object work relevant for policymaking. This may not always occur through normative modes of operation, but the group shall explore questions of policy in a more neutral, explorative tone. This comes about through provision of EU-wide coverage of bio-objects and their central role as a generative force behind much of today’s vital politics.

## Which generative relations matter?

As to where to start exploring bio-objects and their network generation capacities, we suggest that even if life is ubiquitous on our planet (and perhaps elsewhere), facts that matter and theories are not. Theories and interventions addressing any*thing* we call life are always specific, and in that specificity they are actually quite rare. A rarefying principle holds true for the concept of the “bio-object:” not everything is considered to be life, or at least life that matters so much that it becomes stabilized as an object to be represented, circulated, re-formed, and perhaps rebuilt (Box 2).

Box 2 **Bio-objects and generative relations**

Our tentative scoping of bio-objects as empirical entities focuses on products of biological manufacture enabled by technologies of life – in particular, technologies and action enabling (re)new(ed) objects of life, such as laboratory and biomedical practices coupled with bio-informatics/computing; collection, conservation, and biobanking technologies targeting organic matter; manipulation, remodeling, and rebuilding techniques such as genetic engineering and synthetic biology; and, most centrally, the generative relationship work (individual as well as institutional) that goes into the objectification of specific instances of life.

This means that bio-*objects* are not vaguely defined *things*. Instead of being just any*thing*, bio-objects have a presence and a particular relation to life and processes of objectification. Asserting this does not mean recourse to an essentializing notion of “object.” Rather, it hints both at the objects’ material and socially ordered form; it is distinct from being a mere “thing” without internal (material) or social (exterior/embedded) order. These two modes of ordering are always relational processes that can be traced in/to particular situations. In their materiality, objects are both generative of and constituted through a set of empirical relations – whether inside or outside the laboratory.

Studying bio-objects as more or less stable objects means that we take them as materialized relational effects occurring in different social circuits. In these relationship networks, bio-objects become, for example:

- living material as traded goods in global/local bio-economies. Here, hopes, hype, expectations, and larger bio-economic fields of circulation generate particular biosocial spheres of action and hold their central objects as generative to biovalue (2) and sources for accumulated biocapital (3); and

- functional re-creations of (part of) human or animal bodies, pointing to “regen-“, “trans-“, “synth-“, and other processes, along with new relations within and beyond regenerated and reconfigured bodies.

Within these spheres and their constitutive empirical circuits, bio-objects can also become mediators/generators of new relations, namely:

- as objects mediating/generating new kinds of social forms and socia(bi)lity. Here the bio-objects are simultaneously understood as embodied material records of biosocial (4) relations and as their material condition of possibility, tying in with a number of relations beyond economic exchange (5);

- as objects of political debate denoting a central symbolic position within a dispute, pointing to questions of ethics and good governance of life (here, bio-objects become central tokens in *contested* social relations: descriptions of a destabilized social fabric, as well as processes used in attempts at resolution) (6); and

- as objects in an unidentifiable social role outside conventional relations, generating hybrid and/or subversive categories of life. These bio-objects become a subject of ethical/political debate when introduced in the societal realm, as they challenge the whole network of relations at the root of the cosmology of a community.

For Working Group 3, the notion of bio-objects is, therefore, proposed as a conceptual starting point for exploration of the various objects of life in their constitutive relations. While the concept is not yet fully formed – it is open to change through research evidence – it is a useful one, for bio-objects encompass a large number of materialities and accompanying discourses, and, most importantly, a bio-object implies a new form of operationalization of *a* living thing that becomes an object through specific mattering-relations. For analysis, this provides a novel positivity, as the idea of life does not demand recourse to the imagined universality of “life itself,” equivalent to an unspecified “thing” or an assumed vitality lurking behind and beyond living objects.
